# NF-κB p50 subunit knockout impairs late LTP and alters long term memory in the mouse hippocampus

**DOI:** 10.1186/1471-2202-13-45

**Published:** 2012-07-11

**Authors:** Kensuke Oikawa, Gary L Odero, Eric Platt, Melanie Neuendorff, Avril Hatherell, Michael J Bernstein, Benedict C Albensi

**Affiliations:** 1 Div’n. of Neurodegenerative Disorders, St. Boniface Hospital Research, Winnipeg, MB, Canada; 2 Dept. of Psychological and Social Sciences, Pennsylvania State University, Abington, PA, USA; 3 Dept. of Pharmacology & Therapeutics, University of Manitoba, Winnipeg, MB, Canada; 4 St. Boniface Research Ctr., 351 Tache Ave. / R4050, Winnipeg, MB, R2H 2A6, Canada

**Keywords:** Hippocampus, NF-kappa B, Water maze, LTP, Transcription, Memory

## Abstract

**Background:**

Nuclear factor kappa B (NF-κB) is a transcription factor typically expressed with two specific subunits (p50, p65). Investigators have reported that NF-κB is activated during the induction of *in vitro* long term potentiation (LTP), a paradigm of synaptic plasticity and correlate of memory, suggesting that NF-κB may be necessary for some aspects of memory encoding. Furthermore, NF-κB has been implicated as a potential requirement in behavioral tests of memory. Unfortunately, very little work has been done to explore the effects of deleting specific NF-κB subunits on memory. Studies have shown that NF-κB p50 subunit deletion (p50^−/−^) leads to memory deficits, however some recent studies suggest the contrary where p50^−/−^ mice show enhanced memory in the Morris water maze (MWM). To more critically explore the role of the NF-κB p50 subunit in synaptic plasticity and memory, we assessed long term spatial memory *in vivo* using the MWM, and synaptic plasticity *in vitro* utilizing high frequency stimuli capable of eliciting LTP in slices from the hippocampus of NF-κB p50^−/−^ versus their controls (p50^+/+^).

**Results:**

We found that the lack of the NF-κB p50 subunit led to significant decreases in late LTP and in selective but significant alterations in MWM tests (i.e., some improvements during acquisition, but deficits during retention).

**Conclusions:**

These results support the hypothesis that the NF-κ p50 subunit is required in long term spatial memory in the hippocampus.

## Background

A growing body of literature supports a role for NF-κB in synaptic plasticity and memory [1-10]. Long term potentiation (LTP), a paradigm used to measure synaptic function and a molecular correlate of memory, along with behavioral tests of memory, are routinely used to assess hippocampal-dependent alterations in synaptic plasticity and memory [11-19]. Early evidence to support NF-κB’s expression following hippocampal LTP, was discovered in rats *in vivo*[2]. Also, in the Crab, Chasmagnathus, κB-like DNA-binding activity was found enhanced after long term habitation [20]. In addition, Albensi and Mattson [5] showed impairments in mouse hippocampal LTP when NF-κB activity was blocked with κB decoy DNA. Yeh et al. [21] also showed that histone deacetylase (HDAC) inhibitors enhanced long term memory in the rat amygdala and that these effects could be attenuated with κB decoy DNA administration.

The use of specific knockout mice has allowed further clarification concerning NF-κB’s role in memory, although little work has been accomplished along these lines in spite of the importance. For example, Kassed et al [3] demonstrated that lack of the NF-κB p50 subunit (p50^−/−^) impaired memory in an *in vivo* active avoidance paradigm. In a later study by Kassed and colleagues [22], NF-κB p50^−/−^ mice also showed reduced anxiety-like behaviors in open field and elevated plus maze experiments. More recently, Denis-Donini et al. [6] demonstrated selective defects in short-term spatial memory performance in p50^−/−^ mice in a Y maze, but without impairment of long-term spatial memory using the Morris water maze (MWM). However, studies in 2010 by Lehmann et al. [23] suggested that p50^−/−^ mice learned more rapidly when attempting to find the hidden platform in the MWM than did wild type mice. Given these contradictory findings, questions still remain concerning specific roles for NF-κB in memory especially in a context of the NF-κB p50 subunit.

The objective of this study was to investigate the role of NF-κB in both synaptic plasticity and long term spatial memory in p50^−/−^ mice (also known as NF-κB1 knockouts). These mice were used to test if the absence of p50 negatively affected *in vivo* MWM performance and *in vitro* LTP responses - in the same animals. To date, no *in vitro* LTP experiments have been performed in p50^−/−^ hippocampal slices and no studies have attempted serial *in vivo* and *in vitro* tests in the same animals. Our results show that the absence of the NF-κB p50 subunit decreases late LTP, but not early phases of LTP and selectively alters long term spatial memory in p50^−/−^mice (differences in acquisition vs. retention phases of the MWM).

## Methods

### Animals

Two month-old NF-κB p50 male knockout mice (B6;129P2-Nfkb1^tm1Bal^/J) (p50 ^−/−^) and the recommended male controls (B6129PF2/J) (p50 ^+/+^) were purchased from Jackson Laboratory (Bar Harbor, ME, USA). Targeted disruption of the NF-κB p50 subunit has been described in detail previously [24]. The p50^−/−^ mice were homozygous for the deletion of the NF-κB p50 subunit, rendering them incapable of producing the p50 protein. Mice homozygous for the Nfkb1^tm1Bal^ targeted mutation are viable and fertile. Homozygous mutant mice exhibit defective B cell responses, defective responses to infection, and also defects in basal and specific antibody production. Mice were maintained on a 12 hour light/12 hour dark cycle at room temperature (22°C) in the pathogen-free animal facility at the St. Boniface Research Centre. Mice were tested at 4 months of age. The University of Manitoba Animal Care and Use Committee approved all procedures, which conformed to the Guide to the Care and Use of Experimental Animals, published by the Canadian Council for Animal Care.

### Morris water maze

MWM methods have been described previously by Albensi and colleagues [25-27]. Hippocampal-dependent spatial memory was assessed in a standard MWM, which consisted of an 81 cm circular pool, filled with water (24-25°C) and made opaque (white in color) with powdered milk. Visual cues were positioned equidistant above water level and unwanted extra-maze cues were blocked with a dark curtain. A non-visible escape platform, 7 cm in diameter, was submerged ~5 mm below the water surface in the center of the designated target quadrant. Several parameters (e.g., escape latency, swim speed, path length, time in target quadrant, number of passes over the target, and search strategies) were measured in two phases. For the acquisition phase, mice were given < 90 seconds to find the hidden platform and were required to remain seated on the platform for 10 seconds after which the mice were returned to their home cage. In this phase, each animal was tested for 4 trials/day (4 trials = 1 block = 1 day) over 7 consecutive days. Live video was recorded for each trial using a standard tracking system (Videomex, Columbus Instruments, Columbus, OH, USA). The annulus crossing index (ACI), a measure of how many times the subject crosses over the target, was calculated by subtracting the mean number of passes over three alternative sites in the other quadrants from the number of passes over the platform site in the target quadrant. A positive index is indicative of a selective focal search of the previous platform position, an index approximating zero is reflective of a non-specific search, and a negative index is representative of a selective focal search in the quadrants apart from the target quadrant. Search strategies were also assigned for every trial of every block of the acquisition phase, as previously defined by Brody and Holtzman [28]. MWM search strategy analysis has been used by several investigators previously [28-30]. In the Brody and Holtzman classification scheme, up to nine different strategies are coded (by an investigator blinded to strain type), and are then categorized into three broad categories (i.e., Spatial: spatial direct, spatial indirect, focal correct; Nonspatial systematic: scanning, random, focal incorrect; Repetitive looping: chaining, peripheral looping, circling). One strategy that best described the majority of the swim path was assigned to each trial. The retention phase began 24 hours after the last block of the acquisition phase (i.e., day 8). For the retention phase (also known as the probe trial), the platform was removed from the pool and each mouse was given a maximum of 2 minutes to search for the position of the missing platform. During the retention phase, each animal was tested for 4 trials/day for 3 consecutive days.

### Hippocampal slice preparation and electrophysiology

Electrophysiological techniques with brain slices have been described previously [5,25,31]. Mice, which were first used in the MWM experiments (above), were killed by decapitation following anesthesia with isoflurane. The brain was rapidly removed and placed in ice-cold (4-6°C) low calcium artificial cerebrospinal fluid (aCSF), containing (in mM): NaCl, 124; KCl, 3; KH_2_PO_4_, 1.25; MgCl_2_, 1.4; CaCl_2_, 1; NaHCO_3_, 26; glucose, 10. The aCSF was equilibrated with 95% O_2_/ 5% CO_2_ (pH 7.4) throughout the dissection. The left hippocampus was dissected free and sectioned (350 μm thick) using a McIlwain tissue chopper (TC752, Campden Instr., Lafayette, IN). Slices were made perpendicular to the longitudinal axis of the hippocampus, and those used were selected from the middle portion. One slice was used for LTP, one slice was used for paired pulse experiments, and one slice was used for input output curves, but only one slice from each animal was used for each experiment. Remaining slices were used for Western blotting (see below). Slices were collected in an incubation chamber with oxygenated aCSF (95% O_2_/ 5% CO_2_) at room temperature (~21°C). The incubation temperature was gradually increased up to 31-32°C. After a 60 minute recovery, slices were then gently transferred to another incubation chamber or the recording chamber using a standard recording buffer, containing (in mM): NaCl, 124; KCl, 3; KH_2_PO_4_, 1.25; MgCl_2_, 1.4; CaCl_2_, 2; NaHCO_3_, 26; glucose, 10. All aCSF buffers were equilibrated with 95% O_2_/ 5% CO_2_ (pH 7.4).

Tungsten stimulating electrodes were placed on the Schaffer collaterals in the CA1 subregion of hippocampal slices. Input–output curves were generated at the beginning of each experiment to determine maximal and half-maximal responses and voltage settings (maximum response was also determined at the end of the experiment). The voltage was then set to evoke an EPSP response that was approximately half-maximal in amplitude. To evoke orthodromic field excitatory postsynaptic potentials (fEPSPs or EPSPs), monophasic test pulses were delivered to the slice every 60 seconds (Grass S48 stimulator, Warwick, RI) with a stimulus duration of 0.1 ms. In some slices, paired pulse stimulation in the CA1 (50 ms interstimulus interval) was also conducted. Recording was accomplished with an AxoClamp 2B amplifier (Axon Instr., Foster City, CA) in continuous current clamp bridge mode. EPSPs were obtained using glass microelectrodes (2.3 Mohms) filled with recording buffer, which were generated in a standard recording chamber - submerged configuration (RC-27 L, Warner Inst., Hamden, CT) at 31-32°C. EPSP responses were recorded from the CA1 dendritic arbor. EPSP slope values were calculated by measuring the rise/run (i.e., 10-90% of trace). Responses were amplified (gain 50x) low-pass filtered at 6 kHz and digitized (20 kHz) (DIGIDATA 1322A, Axon Instr., Foster City, CA). High frequency stimulation (HFS) for inducing LTP responses consisted of 3 trains of 100 Hz (separated by 0.5 seconds) with each having a one second duration. A stable control baseline period using test pulses was established for 30 minutes before HFS. Test pulse responses were then followed for ~6.5 hours after HFS. Data were acquired with Clampex 9.2, (Axon Instr., Foster City, CA) analyzed initially with Clampfit 9.2 (Axon Instr., Foster City, CA).

### Western blot analysis

The NuPAGE®Bis-Tris, 4-12% Mini Gel (1 mm thickness - Invitrogen) was used for electrophoresis of proteins. Samples were obtained by slicing the hippocampus into 350 μM sections (see above). Extracted protein samples (~40-60 μg) were separated on NuPAGE gels at constant voltage (100 V) for 150 minutes at 4°C following the NuPAGE® technical guide. Proteins separated on a NuPAGE gel were transferred onto nitrocellulose membrane using the Trans-Blot SD Semi- Dry Electrophoretic Transfer Cell (Bio-Rad) following the manufacturer’s instruction. Transfer was carried out at constant voltage (20 V) for 35 minutes at room temperature. After blocking the membrane with 5% skim-milk in TBS solution containing 0.1% Tween-Twenty (TBST) for 1 hour at room temperature, it was incubated with rabbit anti-NFkB p105/p50 (Cell Signalling Technology), diluted 1:500 in 5% skim-milk TBST, overnight with gentle rocking at 4°C. The membrane was washed 3 times in 30 minutes (3 × 10 minute washes) with TBST. After the last wash, the membrane was incubated with secondary antibody, goat anti-rabbit with horseradish peroxidase (HRP) from Epitomics, Inc, diluted in 5% skim-milk TSBT 1:2500 at room temperature for 1 hour. The membrane was then washed with TBST (3 × 110 minutes). The chemiluminescence signal was visualized using the ECL-Plus detection system (GE Health Care Bio-Sciences) following the manufacturer’s instruction. Following visualization, the membrane was washed with TBST (3 × 10 minutes), and then blocked for 1 hour at room temperature with 5% skim-milk TBST. After blocking, the membrane was incubated with rabbit anti-Actin 1:5000 (Sigma-Aldrich), overnight at 4°C with gentle rocking. The membrane was washed (3 × 10 minutes) with TBST. After the last wash, the membrane was incubated with secondary antibody, goat anti-rabbit with horseradish peroxidase (HRP) from Epitomics, Inc, diluted in 5% skim-milk TSBT 1:2500 at room temperature for 1 hour. The membrane was then washed with TBST (3 × 10 minutes). The chemiluminescence signal was visualized using the ECL-Plus detection system (GE Health Care Bio-Sciences) following the manufacturer’s instructions.

### Data handling and statistical analyses

Analyses of variance (ANOVA) were used to assess main effects and interactions. For MWM experiments, mixed model ANOVAs were used (2 strains × 7 days). Post hoc, simple effect t-tests were applied when a significant interaction was found between factors. Other t-tests were applied when appropriate. For the search strategy assessment, comparisons of strategy use between strains were accomplished by Chi-square analysis. For electrophysiological experiments, only one slice was used from each animal for each experiment, which eliminates potential slice averaging bias and/or imbalanced slice to animal ratios. The magnitude of LTP in a single slice was determined by comparing the mean EPSP response at various 5 minute intervals (i.e., 1, 2, 3, 4, 5, and 6 hours) post-tetanization with the mean EPSP response for a 5 min period just before tetanization (e.g., at 26–30 minutes). Electrophysiological input–output curves and LTP results were analyzed by mixed model ANOVAs (strain × time) and followed by simple effect t-tests when appropriate. Paired pulse ratios were created by dividing the second EPSP response slope values by the first EPSP response values (i.e., EPSP2/EPSP1). SPSS software 19.0 (SPSS Inc., Chicago, IL, USA) was used for all analyses. P < 0.05 was considered significant. All error bars in figures are + S.E.M.

## Results

### Morris water maze performance in NF-κB p50 Mice

**Figure 1:**
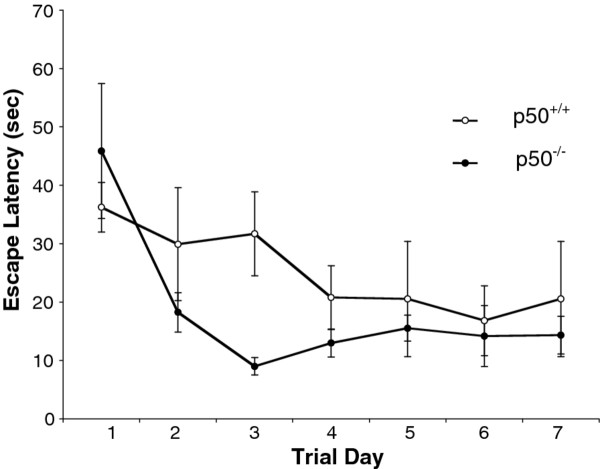
**Escape latency during Morris water maze testing.** Escape latency was measured in p50 mice during the acquisition phase, where both groups significantly decreased (P < 0.001) their escape latency times over 7 days of testing (p50^+/+^: from mean 36 s to 20 s; p50^−/−^ mean 46 s to 14 s), but there was no statistical difference (P = 0.39) between p50^−/−^ and p50^+/+^ mice over the 7 day period. A trend is seen on day 3 where p50^−/−^mice show shorter escape times.

In order to assess long term memory in intact animals, we tested p50^+/+^ and p50^−/−^ mice (n = 6 for each strain) in the MWM, a behavioral paradigm designed for assessing hippocampal-dependent spatial memory. We performed a 2 strain (p50^−/−^, p50^+/+^) × 7 day (days 1 through 7) mixed model ANOVA with repeated measures on the second factor to examine the effect of several days of training and potential strain differences. Figure 1 shows the change in escape latency for both strains during the 7 day acquisition phase. In this experiment, the results show a significant main effect for days related to training for all p50 mice [F(6,60) = 5.47; P < 0.001], that is a general decrease in escape latency was seen as training time progressed. However, we found no significant main effect between the p50^−/−^ and p50^+/+^ mice (P = 0.39) nor any interaction over the 7 day period [F(1,10) = 0.002; P = 0.96]. Interestingly, a visual inspection of this figure on day 3, does show a non-significant enhancement in performance for the p50^−/−^ mice (see discussion).

**Figure 2:**
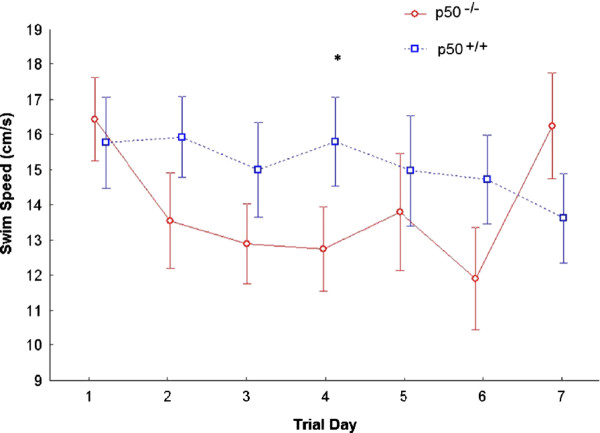
**Swim speed assessment during Morris water maze testing.** Swim speed was measured during the acquisition phase in p50 mice. There was no significant change in swim speed over the 7 days for both groups (P = 0.05). However, post hoc testing showed a significant difference (*P = 0.025) between strains on day 4 of testing where p50^−/−^ mice swam slower than control mice.

**Figure 3:**
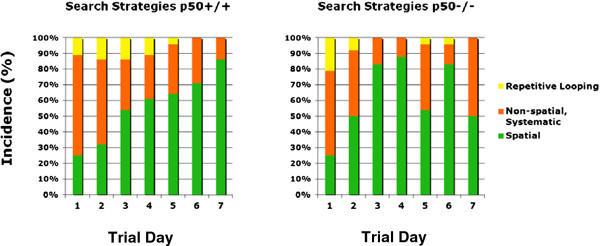
**Search strategy assignments during Morris water maze testing.** Search strategies were assigned for each mouse during the acquisition phase. Search strategies were categorized into three main groups: Spatial, Non-spatial Systematic, and Peripheral Looping. The data (right panel) show a disturbance in the formation of spatial memory in the p50^−/−^ mice as represented by fluctuations in their usage of spatial search strategies. p50^+/+^ mice, on the other hand (left panel) show an expected gradual and steady increase in the proficient use of this hippocampal-dependent spatial strategy over time, which is consistent with several other studies. Chi-square tests showed several significant associations (P < 0.05) between strain and the adopted search strategies (days 2, 3, 4, and 7).

We also measured swim speed (Figure 2) during the acquisition phase. We performed a 2 strain × 7 day mixed model ANOVA with repeated measures on the second factor on the swim speed scores after data reduction was performed. The results yielded no significant main effect of day [F(6,66) = 2.24, P = 0.05]. Also there was no main effect of strain on swim speed [F(1,11) = 1.78, P = 0.21]. However, a trend was seen such that as the days progressed, the swim speed generally decreased. This was associated with a significant interaction between strain and day, indicating that swim speed varied as a function of the day and strain of mouse [F(6,66) = 3.30; P = 0.007]. Simple effects analyses revealed that while strain did not result in significant differences on days 1,2,3,5,6, or 7 (all P’s > 0.70), the strains did differ at day 4 (P = 0.025); the p50^−/−^ mouse strain (M =12.85, SD = 1.12) had a significantly slower swim speed than did the p50^+/+^ strain (M = 16.01, SD = 2.77). Figure 3 shows search strategy graphs generated from the acquisition phase. We calculated the frequencies associated with the adoption of each strategy each day for each strain and also applied Chi-square analyses for statistical assessment (Table 1). We found that p50^+/+^ mice used a mixture of strategies on the first day, including spatial (25%), non-spatial (64%), and repetitive looping (11%) and then showed a progressive increase (day 2, 32%; day 3, 54%; day 4, 61%; day 5, 64%; day 6, 71%; day 7, 86%) in the use of spatial strategies over the rest of the 7 day acquisition phase, which is identical to the pattern of control search strategy data from several other studies [25,28,32]. Whereas, p50^−/−^ mice showed daily fluctuations in their adoption of spatial search strategies (day 1, 25%; day 2, 50%; day 3, 83%; day 4, 88%; day 5, 54%; day 6, 83%; day 7, 50%), suggesting p50^−/−^ mice had a disturbance in the formation of spatial memory. Quantitative analysis of these data using Chi-square tests showed several significant associations between strain and the adopted search strategies. For day 1 (χ^2^ = 3.97; P = 0.137), day 5 (χ^2^ = 5.90; P = 0.052), and day 6 (χ^2^ = 5.98; P = 0.050) there was no significant association, but for day 2, there was a significant association (i.e., P < 0.05) between strain and strategy (χ^2^ = 7.09; P = 0.029) and also for day 3 (χ^2^ = 24.7; P < 0.001), day 4 (χ^2^ = 22.3; P < 0.001), and day 7 (χ^2^ = 33.8; P < 0.001). However, one should notice that on days 5 and 6, significance was almost reached (day 5, P = 0.052; day 6, P = 0.050).

**Table 1:** **Summary of*in vivo*search strategy data: Percentage of use over time during acquisition training.**

Strain	**Strategy**	**Day 1**	**Day 2**	**Day 3**	**Day 4**	**Day 5**	**Day 6**	**Day 7**
NF-κB p50+/+ mice	Spatial	25	32	54	61	64	71	86
	Non-Spatial	64	54	32	28	32	29	14
	Repetitive Looping	11	14	14	11	4	0	0
NF-κB p50−/− mice	Spatial	25	50	83	88	54	83	50
	Non-Spatial	54	42	17	12	42	13	50
	Repetitive Looping	21	8	0	0	4	4	0

**Figure 4:**
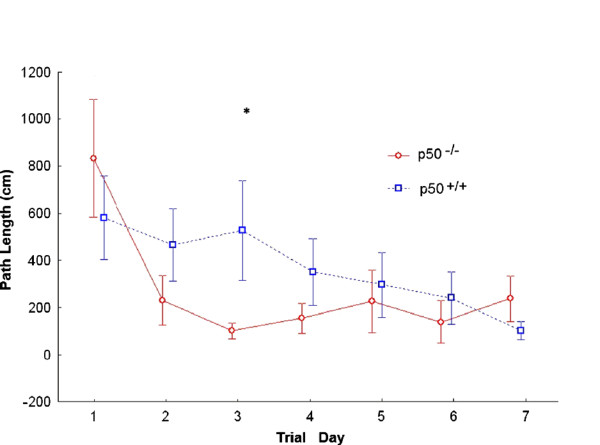
**Path length determination during Morris water maze testing.** Path lengths (i.e., distance travelled) were measured for p50 mice during the acquisition phase. Over this period, a significant effect was seen for both groups over time (P < 0.001). Post hoc analyses also showed a significant difference (*P = 0.014) between strains on day 3 where the p50^−/−^ mice showed a shorter distance travelled.

Figure 4 shows path length. We performed a 2 strain × 7 day mixed model ANOVA with repeated measures on the second factor on the path length scores after data reduction was performed. The results yielded a significant main effect of day, such that as the days progressed, the path length was reduced [F(6,66) = 7.341; P < 0.001]. This was qualified, however, by a significant interaction between strain and day, indicating that path length varied as a function of the day and strain of mouse [F(6,66) = 2.718, P = 0.020]. Simple effects analyses revealed that while mouse strain did not result in significant differences at days 1,2,4,5,6,7 (all P’s > 0.100), the strains did differ at day 3 (P = 0.014); the p50^−/−^ strain (M = 116.41; SD = 34.32) had a significantly shorter path length than did the p50^+/+^ strain (M = 535.99; SD = 350.32). Additional analyses of the differences between days as a function of strain revealed significant differences as well. For the p50^−/−^ strain, while day 1 path length (M = 757.50, SD = 443.83) was significantly higher than all other days (all P’s < 0.02), none of the other days differed from each other (all P’s > 0.23). A somewhat differed pattern emerged for the p50^+/+^ strain; here, a more gradual decrease in path length seems to have occurred, with differences between days occurring throughout the advancing days.

**Figure 5:**
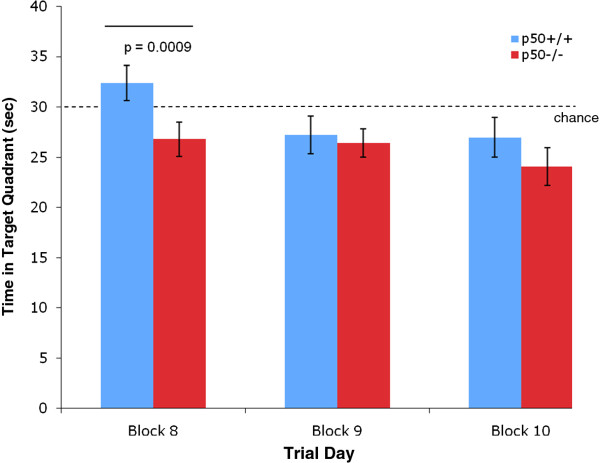
**Time in target quadrant during Morris water maze testing.** The time in target quadrant was measured during the three day retention phase. The p50^−/−^ mice showed a highly significant (P = 0.009) decrease relative to the p50^+/+^ mice for this measurement on the 8^th^ day of testing (i.e., first day of the retention phase). The experiment was conducted for 2 minutes (120 s) and so the dashed line drawn at 30 seconds indicates chance levels.

**Figure 6:**
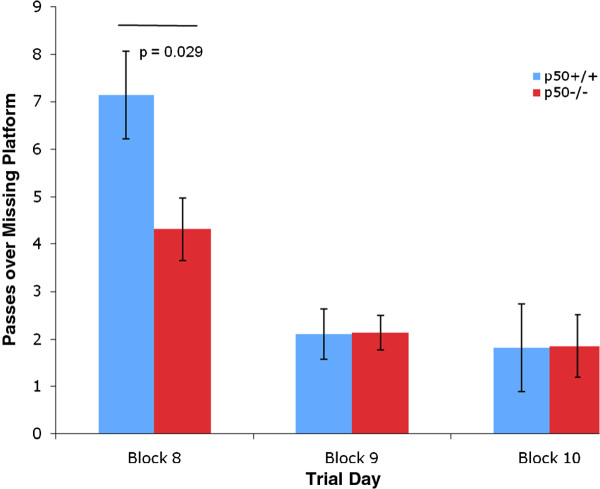
**Passes over the missing platform during Morris water maze testing.** Passes over the missing platform were measured during the retention phase. p50^−/−^ mice were found to pass over the missing platform significantly less frequently (P = 0.029) than p50^+/+^ mice on day 8.

**Figure 7:**
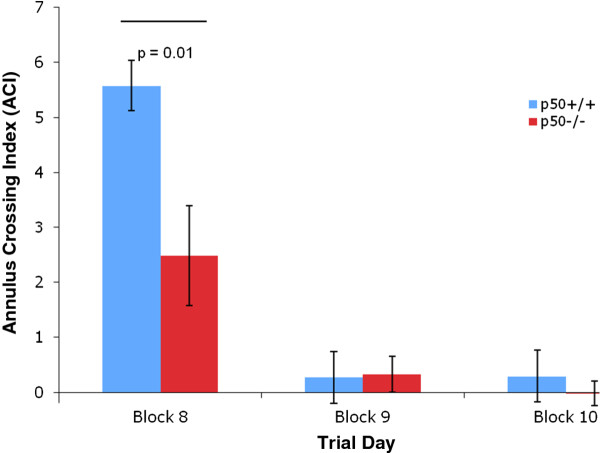
**Annulus crossing index during Morris water maze testing.** The annulus crossing index was measured during the retention phase. The p50^−/−^ mice showed a significant (P = 0.01) decrease relative to the p50^+/+^ mice.

In addition, several measures were made during the retention phase, which began on day 8. Identical to the procedures used in other labs, the target platform was removed during this 3 day phase. We measured the time in the target quadrant during retention and found a significant decrease (P < 0.01) in this parameter in p50^−/−^ mice (Figure 5) on day 8. In addition, we measured the number of passes over the missing platform (Figure 6) and calculated the annulus crossing index (ACI), which indicated that p50^−/−^ mice showed a highly significant (P < 0.01) impairment (Figure 7) in spatial bias for the platform position in the target quadrant relative to the p50^+/+^ mice. In other words, p50^−/−^ mice crossed over the target platform significantly less frequently than the p50^+/+^ mice.

### Basal synaptic transmission in CA1 hippocampal slices

**Figure 8:**
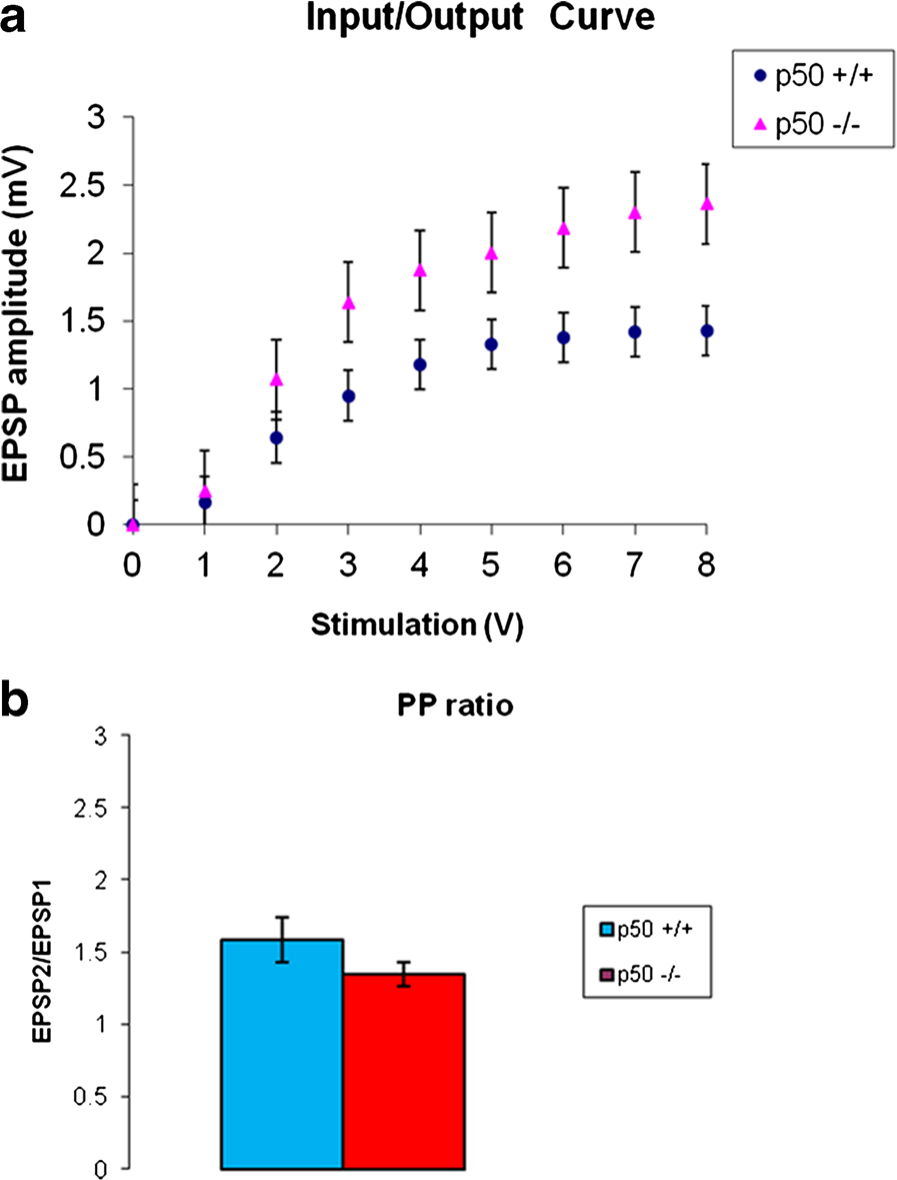
**Basal synaptic transmission in CA1 hippocampal slices.** A: Input–output relations were measured as a function of increased voltage. We found no significant difference between p50^−/−^ and p50^+/+^ strains (P = 0.109) when assessing input–output curves. However, we did find a significant main effect of voltage on EPSP amplitude response (P < 0.001). In addition, we found a significant interaction between strain and stimulation voltage (P = 0.004). B: We also compared paired-pulse responses, an indicator of presynaptic function, and found no significant difference (P = 0.34) between strains using an interstimulus interval of 50 ms

We evaluated input–output relations after using test pulses in the CA1 subregion of the hippocampus to determine if overall neuronal excitability levels were different in p50^−/−^ vs. p50^+/+^ slices (n = 6 for each strain) as one increased voltage. We found no main effect of strain on EPSP response (amplitude). In other words, we found no significant difference between p50^−/−^ and p50^+/+^ strains [F(1,10) = 3.104; P = 0.109] when assessing input–output curves (Figure 8A).

However, we did find a significant main effect of voltage on EPSP response [F(8,80) = 60.14; P < 0.001]. In addition, we found a significant interaction between strain and stimulation voltage [F(8,80) = 3.109; P = 0.004]. A post hoc simple effect t-test subsequently showed a significant difference between both strains at 2 volts (P = 0.02) and at 8 volts (P = 0.04).

We also assessed paired pulse responses (50 ms interstimulus interval), which is an indicator of potential changes in presynaptic activity. We used the independent sample t-test to compare paired pulse responses between the two strains (n = 6 for each strain). In this case, we found (Figure 8B) that there was no significant difference between paired pulse responses (P = 0.34).

### LTP in CA1 hippocampal slices

**Figure 9:**
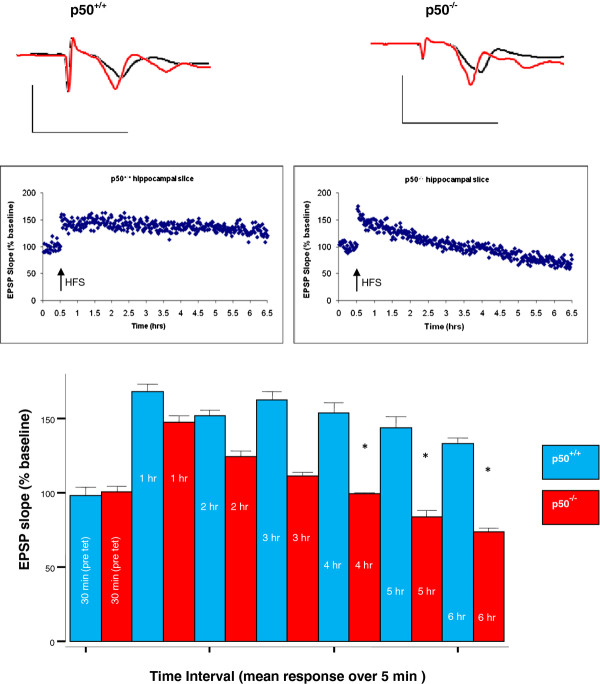
**LTP evaluation in hippocampal slices.** LTP was reliably induced in both groups in CA1 hippocampus immediately after high frequency stimulation (HFS - black arrow). Early LTP time points (1, 2, and 3 hours - 5 min sampling intervals) show no significant difference in LTP responses between the two groups (P > 0.05). However, a significant decrease (*P < 0.05) was found in EPSP slope in the p50^−/−^ hippocampal slices (triangles) vs. p50^+/+^ (circles) slices at 4, 5, and 6 hour time points (5 m sampling intervals) indicating deficits in late LTP. Scale bar: 2 mV, 10 ms.

Electrophysiology experiments using HFS were conducted in CA1 hippocampal slices from NF-κB p50 mice (p50^+/+^ vs. 50^−/−^) in order to assess LTP responses. HFS has been previously shown by our lab [5,25,31] and many other labs [33] to reliably induce LTP, an experimental paradigm of synaptic plasticity and suspected correlate of memory. The mice used for these electrophysiological experiments (n = 6 for each strain) were the same ones that were described above in our MWM experiments. We used mixed model ANOVA to look for main effects of strain on LTP responses and of time on LTP responses. We found (Figure 9) a significant main effect for time on LTP responses [F(6,60) = 9.20; P < 0.001]. We also found a significant interaction [F(6,60) = 4.62; P = 0.001] between time and strain. Post hoc simple effect t-tests subsequently showed several significant differences between time intervals and between strains at specific times following HFS. We tested slices for more than 3 hours (i.e. up to 6 hrs) post HFS since three hours or more coincides with the onset of late LTP, which is associated with the persistent regulation of gene expression and the initiation of long term memory, whereas LTP responses before 3 hours is generally protein-synthesis independent [33,34]. We found that both strains showed significant increases in LTP at 1 hour post HFS as compared to their respective control baseline EPSP values (P < 0.001 for p50^+/+^ and P = 0.005 for p50^−/−^). In addition, we found that p50^−/−^ slices showed significantly lower LTP responses at 4 hours (P = 0.047), 5 hours (P = 0.038), and 6 hours (P = 0.045) post HFS as compared to LTP responses in p50^+/+^ slices; but this was not true at earlier time points (P > 0.05). However, some trend differences were seen between strains at early LTP time points (i.e., within the first ten minutes), but as noted, these values were not statistically significant. Overall, not only did p50^−/−^ LTP responses decay over time, they also reached values lower than baseline at 5 and 6 hours post HFS.

### Expression of NF-κB p50 subunit

**Figure 10:**
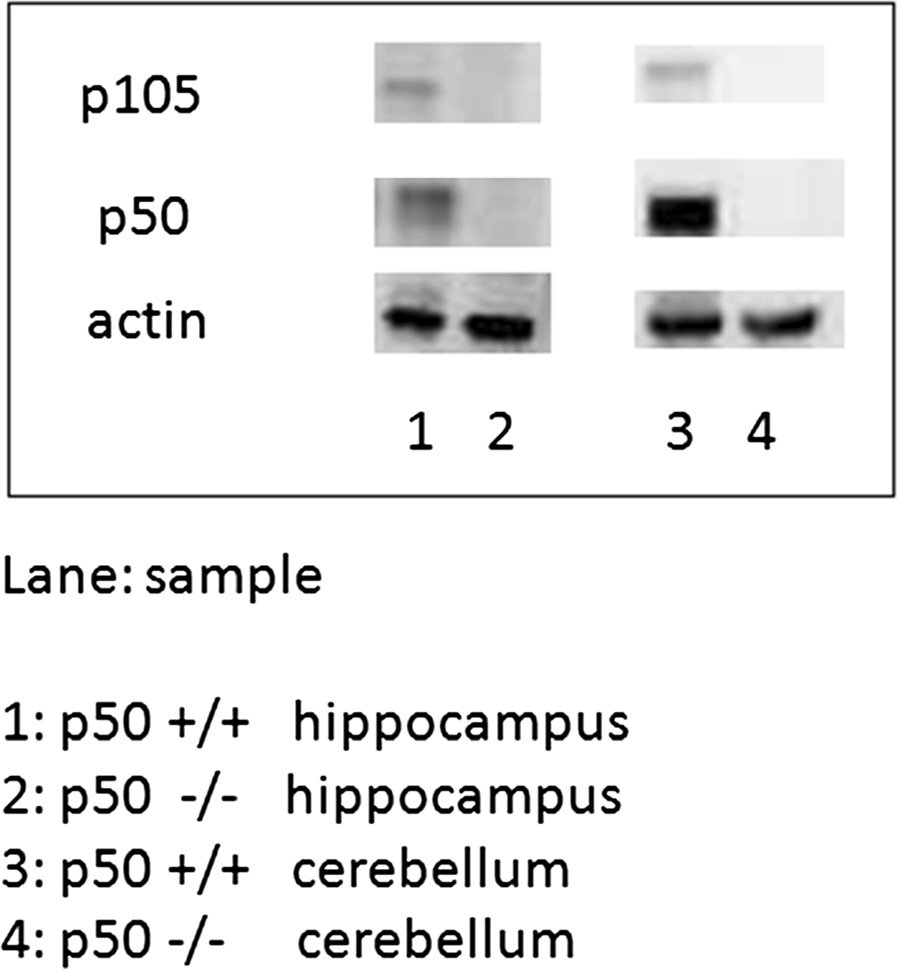
**NF-κB subunit expression in hippocampal and cerebellum NF-κB p50 mouse samples.** Representative Western blots are shown indicating that NF-κB p50 subunit expression was relatively absent in hippocampal and cerebellum samples from p50^−/−^ mice following LTP experiments.

In order to verify that the NF-κB p50 subunit protein was knocked out in NF-κB p50^−/−^ brain regions, we assessed NF-κB p50 subunit expression using Western blotting techniques in homogenized hippocampal and cerebellum samples taken from NF-κB p50^+/+^ and p50^−/−^ mice following LTP experiments (n = 2–3 animals for each brain region and for each strain sampled). We found that NF-κB p50 subunit expression was significantly reduced (P < 0.05) and was virtually absent in both hippocampal and cerebellum regions (Figure 10) taken from NF-κB p50^−/−^mice as compared to the same regions taken from NF-κB p50^+/+^ mice. In addition, we observed NF-κB p105 subunit expression (NF-κB p105 subunit undergoes cotranslational processing by the 26 S proteasome to produce the NF-κB p50 kD protein) in hippocampal and cerebellum samples taken from NF-κB p50^+/+^ mice, but not in NF-κB p50^−/−^ mice. These results confirm that the NF-κB p50^−/−^ mice used in these experiments lacked the NF-κB p50 subunit in the regions tested, which has direct implications for the MWM and electrophysiology experiments discussed above.

## Discussion

We previously demonstrated [5], as have others, that NF-κB plays a role in synaptic plasticity and memory. In addition, two recent investigations using knockout mice have attempted to look more specifically at how NF-κB p50 subunits might be required for memory. However, these studies presented contradictory findings where p50^−/−^ mice were found in one case [23] to have enhanced long term memory and in the other case was shown to have no change in long term memory [6]. In general, the data here support our prior work, which suggest NF-κB is required for both synaptic plasticity and memory. We also extend prior findings where we found mixed results with MWM performance in NF-κB p50^−/−^ mice and deficits in experiments in p50^−/−^ hippocampal slices involving late LTP responses. Interestingly, our data collectively show that p50^−/−^ mice may learn faster during acquisition phases on specific days, but have significantly poorer recall ability as compared to p50^+/+^ during the retention phase. In addition, we show that LTP can be reliably induced in both strains, but that at time points past 3 hours, p50^−/−^ hippocampal slices demonstrate significantly lower LTP responses than p50^+/+^ hippocampal slices.

For example, in several MWM assessments conducted during the retention phase, we found deficits in p50^−/−^ mice. However, we did not see any difference in mean escape latency times between p50^−/−^ and p50^+/+^ control mice over the 7 day acquisition phase, which is in agreement with Denis-Donini et al.’s [6] study; but we did observe that p50^−/−^ mice found the platform faster on day 3 (but this finding was not statistically significant). Interestingly, in Lehmann et al.’s [23] recent study, p50^−/−^ mice performed significantly better than p50^+/+^ mice on day 3 (and also on their day 2 and 4). Lehmann et al. also found enhancements in p50^−/−^ mice in other MWM assessments (e.g., preference for training quadrant), but not in Barnes’ maze assessments (Barnes’ maze tests were not conducted by us or in the Denis-Donini et al. study). However, in opposition to our study, Denis-Donini et al., found that p50^−/−^ mice had no deficits in long term spatial memory in any of the behavioral tests used (i.e., the MWM and the Y-maze). Whereas, we found significant impairments in p50^−/−^ mice in other MWM assessments (retention phase) for long term memory (e.g., time in target quadrant, ACI, etc.). It is interesting to note that Denis-Donini et al. did find specific short term memory deficits without learning impairment in p50^−/−^mice. At this time, we cannot fully explain the specific discrepancies amongst these three studies (i.e., Denis-Donini et al., Lehmann et al., and our own). It would be interesting to know if housing differences involving environmental enrichment structures among the three labs played a role. However, there was nothing listed in the methods concerning this aspect in the other studies so this is not known.

In addition, we conducted search strategy assessments, which the other two studies did not. Spatial search strategies are hypothesized by many investigators to be more effective for finding the hidden platform than non spatial strategies or looping strategies [28,32] and are associated with forming a so-called spatial or cognitive map. A tenet of hippocampal function ascribes the hippocampus as a region that can create a spatial map [35,36]. The development of a spatial map for the platform location is reflected by a correct focal search in the target quadrant. In both strains, the use of search strategies changed over the 7 days of training, however, p50^++^ mice showed a progressive increase in the proportion of spatial strategies adopted over this 7 day acquisition phase, whereas p50^−/−^ mice did not. The inconsistent use of spatial search strategies by the p50^−/−^ mice may be related to problems with memory retention in the same group.

In addition, we also found that there was a significant difference on day 4 (but not overall) in swim speed, where p50^−/−^ mice swam slower than control mice. Given this finding, it is tempting to speculate that decreased NF-κB p50 subunit expression in the cerebellum (Figure 10) may be related to swim speed differences in p50^−/−^ mice, but if this is the case, the effect appears to be quite minor. With regard to what search strategies were used on day 4, both groups on that day primarily used spatial strategies (~61% for p50^+/+^ and ~88% for p50^−/−^ mice) and so swim speed changes do not appear related to suboptimal spatial search strategy use.

Additional MWM tests following the 7 day acquisition phase were conducted during a 3 day retention phase (i.e., probe trial without platform), which were performed on days 8, 9, and 10. Assessments on day 8 for ACI, time in target quadrant, and passes over missing platform all indicated significant deficits in the p50^−/−^ mice. These retention deficits in p50^−/−^ mice may be related to the inconsistent use of spatial search strategies discussed above. In contrast, Lehmann et al. found no difference between strains during their probe trials. Collectively, our MWM results appear to generally show that p50^−/−^ mice learn specific aspects of the MWM tests faster than their p50^+/+^ counterparts during acquisition training on days 3 and 4 and that p50^−/−^ mice show deficits during retention trials on day 8. However, in our study the adoption of spatial search strategies, which were preferred by the p50^+/+^ mice, cannot be claimed to have contributed to the enhanced learning of the p50^−/−^ mice during acquisition training.

We also found that LTP was reliably induced in both p50^−/−^ and p50^+/+^ slices, but was significantly different between the two strains in late phases of LTP. In other words, hippocampal slices from p50^−/−^ mice showed early LTP, but late LTP responses were significantly decreased, as compared to p50^+/+^ LTP responses. To our knowledge this is the first report assessing LTP responses in brain slices from p50^−/−^ mice. Collectively these results suggest that deficits in late LTP in the p50^−/−^ mice might be associated with alterations in gene expression since prior studies have shown that late LTP is correlated with gene expression. We also examined aspects of basal synaptic transmission and found no significant effect of strain on EPSP response with regard to input output relations. However, we did find a significant main effect of voltage on EPSP response and found a significant interaction between strain and stimulation voltage at 2 and 8 volts. This small increase in network excitability in p50^−/−^ slices suggests either the involvement of more excitatory synapses and/or increased synaptic efficiency at individual synapses. Since NF-κB activity has been shown to modulate AMPA receptor and/or NMDA receptor transcription, one possibility is that NF-κB p50 knockout had some effect on these receptors that influenced small changes in neuronal excitability. These sort of changes might interfere with normal memory-related hippocampal synaptic function, and might contribute to the memory deficit in the p50^−/−^ mice. However, presynaptic transmission, as measured by paired pulse responses, was unaffected in the p50^−/−^ hippocampal slices at the interstimulus interval (ISI) of 50 ms. Future studies, however, could extend our findings by examining additional ISIs across a larger range.

Mice deficit for other NF-κB subunits have also been tested in past synaptic plasticity and memory studies [7,8,37-40]. For example, c-Rel [7,8] and p65 [39] subunit deletions have been evaluated for their potential role in synaptic plasticity and memory, but their specific contributions are not yet clear. It is known that NF-κB is typically expressed with 3 specific subunits (p50, p65, and IκB), which are associated with an inactivated state. Once activated, IκB is ubiquitinated, and NF-κB dimers (typically p50/p65) move from cytoplasm to nucleus and bind to NF-κB sites in the promoter regions of target genes, and activate their transcription and/or expression. Homodimers of p50 or the p50/p52 heterodimer are thought to function as transcriptional repressors, whereas the remaining combinations of NF-κB dimers (e.g., p65/p50) are thought to act as activators [41]. In our studies, the chronic loss of NF-κB p50 subunit might alter gene regulation such that hippocampal synapses are in an altered state. This altered state may be the reason for the observations we made concerning memory retention deficits and impairments in late LTP.

Our study was novel in several ways, which included using LTP paradigms with p50^−/−^hippocampal slices and serial *in vivo* and *in vitro* testing in the same animals, but there were a few limitations to our experiments which we should note. For example, we did not critically test aspects of short-term memory nor did we use other mazes as did Denis-Donini et al. 2008 and Lehmann et al 2010. In addition, we did not directly examine the mice for potential locomotor deficits, which may have contributed to the low annulus crossing index in p50^−/−^ mice; although there were no obvious impairments in motor function that we noticed during MWM testing and other p50^−/−^ mouse studies have not reported any motor deficits to our knowledge. In fact, our swim speed tests did not indicate any impairment in locomotion in p50^−/−^ mice overall, although there were differences on day 4 where p50^−/−^ appeared to swim slower than controls. Future studies in NF-κB p50 knockout mice could test anxiety-related behaviors in mazes such as the elevated plus maze or the open field test (which was not an objective of this investigation), in order to determine how anxiety might negatively affect spatial memory testing. Some work has been attempted along these lines already [22].

It is of course hoped that future studies will also correlate NF-κB subunit specificity and activity with precise specific parameters that assess synaptic plasticity and memory function. Novel studies are also needed that look at age-dependence and how deficits in NF-κB activity might contribute to memory impairments in Alzheimer’s disease, and other CNS disorders, etc. Given that some preliminary work with NF-κB p50 knockouts have shown exacerbations in neuronal degeneration [3], work in this area would be warranted.

## Conclusions

The NF-κ p50 subunit is required for long term spatial memory in the hippocampus.

## Author’s contributions

BCA conceived and designed the experiments. KO conducted the electrophysiology experiments. GO conducted the Morris water maze experiments. GO, EP, and MN performed the data analysis for the Morris water maze. AH and MN conducted the Western blotting experiments. MJB planned and conducted the statistical analyses with some input from BCA. All authors read and approved the final manuscript.
